# Exploring microRNA Biomarker for Amyotrophic Lateral Sclerosis

**DOI:** 10.3390/ijms19051318

**Published:** 2018-04-28

**Authors:** Y.-h. Taguchi, Hsiuying Wang

**Affiliations:** 1 Department of Physics, Chuo University, Kasuga, Bunkyo-ku, Tokyo 112-855a, Japan; tag@granular.com; 2 Institute of Statistics, National Chiao Tung University, Hsinchu 30010, Taiwan

**Keywords:** amyotrophic lateral sclerosis, biomarker, microRNA

## Abstract

Amyotrophic lateral sclerosis (ALS) is among the severe neuro degenerative diseases that lack widely available effective treatments. As the disease progresses, patients lose the control of voluntary muscles. Although the neuronal degeneration is the cause of this disease, the failure mechanism is still unknown. In order to seek genetic mechanisms that initiate and progress ALS, the association of microRNA (miRNA) expression with this disease was considered. Serum miRNAs from healthy controls, sporadic ALS (sALS), familial ALS (fALS) and ALS mutation carriers were investigated. Principal component analysis (PCA)-based unsupervised feature extraction (FE) was applied to these serum miRNA profiles. As a result, we predict miRNAs that can discriminate patients from healthy controls with high accuracy. Thus, these miRNAs can be potential prognosis miRNA biomarkers for ALS.

## 1. Introduction

Amyotrophic lateral sclerosis (ALS) is a difficult disease to effectively treat [1]. In spite of massive efforts spent for the identification of effective treatments, none could ever be established. There are several reasons for this difficulty. One of the reasons for this difficulty is the lack of genetic background that helps researchers to identify disease-causing genes. There are several different types of ALS including classic, sporadic, and familial, that are classified by their signs, symptoms and whether there is a genetic association or not. The sporadic ALS (sALS) has no family history that unfortunately, accounts for 90% of ALS cases. Even in minor familial ALS (fALS) that has a family history of the disease, there are no definite mutations that can cause ALS. There are many candidate genes [2]; superoxide dismutase type-1 (SOD1) [3], senataxin (SETX) [4], TAR-DNA binding protein (TDP)-43 [5] and chromosome 9 open reading frame 72 (C9ORF72) [6] are some of the well-investigated examples. Nevertheless, most of them were identified based on association studies, and thus, we lack the knowledge about how the mutations cause ALS. The second reason is that ALS is the disease of motor neurons, the removal of which would injure patients. This results in the difficulty for researchers to study the genetic mechanisms in tissue samples that induce ALS. Thus, it is not easy to investigate the mechanism for ALS.

Recently, microRNAs (miRNAs) have been discovered to be potential biomarkers of ALS (such as miR-218) [7]. In this paper, we predict other potential miRNA biomarkers in human sera and identify functions enriched in mRNAs targeted by these predicted miRNA biomarkers. We apply the recently proposed principal component analysis (PCA)-based unsupervised feature extraction (FE) [8,9,10,11] to human serum miRNAs and successfully predict miRNA biomarkers that can discriminate healthy controls from ALS patients.

## 2. Results

### 2.1. Identification of Up/Downregulated miRNAs

The miRNA expression profiles used in this study were downloaded from the miRNA Expression Omnibus (GEO) using GEO ID GSE52917 [12]. The file GSE52917_series_matrix.txt.gz included in “Series matrix” section was used. A total of 53 Affymetrix miRNA 3.0 arrays including serum ncRNA profiles of sporadic and familiar ALS patients and asymptomatic ALS mutation carriers compared to age and gender matched healthy controls is used. There is a total of expression profiles of nine fALS patients (analyzed in six arrays), 18 ALS mutation carriers (analyzed in 12 arrays), 18 sALS patients and 17 controls used.

Patients were considered sporadic cases based on a negative family history. Patients with familial ALS as well as asymptomatic mutation carriers were identified by sequencing of the SOD1, PFN1 or FUS genes or by repeat-primed PCR for detection of C9orf72 mutations [12]. All serum samples drawn from control individuals, patients with ALS and pre-manifest ALS mutation carriers were collected by the same center and processed according to standard procedures within 1 h after blood drawing and stored at −80 °C until further usage. The details of the extraction protocol are available from the database miRNA Expression Omnibus (GEO) using GEO ID GSE52917 [12].

By applying the PCA based unsupervised feature extraction, 107 miRNAs are selected to be related to ALS. After comparing with the results of the original study [12], we predict 27 downregulated miRNAs and 24 upregulated miRNAs (Table 1) in discriminating ALS patients from healthy controls.

### 2.2. Identification of miRNAs Expressed Differentially between ALS Patients and Healthy Controls

There is a total of 51 miRNAs identified in this study. We apply the linear discriminant analysis (LDA) to these selected miRNAs. Table 2 shows the confusion table. Excluding sALS patients, LDA can successfully discriminate healthy controls, ALS mutation carriers and fALS patients. The overall accuracy is 0.66 which is relatively high because there are four classes. This does not always mean that serum miRNAs do not have the ability to discriminate sALS from others, but it means that sALS patients are heterogeneous. In the future, when we can classify sALS into several subclasses, serum miRNAs might have the ability to discriminate them. On the other hand, when sALS patients are excluded (Table 2), accuracy raised up to as large as 0.84. This suggests that serum miRNAs can work as biomarkers between healthy controls, fALS and ALS mutation carriers.

We have successfully identified miRNAs that can discriminate healthy controls from ALS patients. Nevertheless, since the purpose of the present paper is not only to predict miRNA biomarkers, but also to explore the genetic background of ALS, in the next section we discuss identified miRNA in more details from the biological point of view.

## 3. Discussions

### 3.1. Uploading Down/Upregulated miRNAs to DIANA-Mirpath

In this section, to enhance our result that the predicted miRNAs may be potential biomarkers of ALS, we apply an mRNA enrichment analysis to find related Kyoto Encyclopedia of Genes and Genomes (KEGG) pathways. We separately uploaded 27 downregulated miRNAs and 24 upregulated miRNAs in Table 1 to DIANA-mirpath [13] (Table S1) that detects KEGG pathways enriched by the targeted mRNAs of these miRNAs.

### 3.2. KEGG Pathway Enrichment Analysis by DIANA-Mirpath

There are 19 enriched KEGG pathways (Table 3) detected by DIANA-mirpath (Table S1) using the 27 downregulated miRNAs (Table 1). Some of them were previously reported to be related to ALS. Kotni et al. [6] reported that “Extracellular matrix(ECM)-receptor interaction” as well as “Focal adhesion” were enriched in upregulated differentially expressed gene in ALS patients. Phatnani et al. [14] also reported the importance of “ECM-receptor interaction” in ALS. Wu et al. [15] reported the reduction of “adherens junction” protein E-cadherin in ALS mouse model. Lee et al. [16] reported that mammalian sterile 20-like kinase 1 (MST1), which is a core module member of “Hippo signaling pathway”, functions as a key modulator of neurodegeneration in a mouse model of ALS. The relation between ALS and “Transforming growth factor β (TGF-β) signaling pathway” was reported [17]. Especially, "ECM-receptor interaction", "Focal adhesion" and "TGF-β signaling pathway", were identified to be reduced in iPSC-derived motor neurons of patients with C9ALS [2]. "ECM-receptor interaction" as well as "TGF-beta signaling pathway" affect motor neuron also in ALS model mouse [3]. A role for “Ubiquitin-mediated proteolysis” in the pathogenesis of ALS was also reported [18,19]. There are some studies that relate “Protein processing in endoplasmic reticulum” to ALS [20,21,22,23]. “AMP-activated protein kinase (AMPK) signaling pathway” was also reported to be related to ALS [24,25,26]. Thus, the majority of enriched KEGG pathways are related to ALS.

We further investigate KEGG pathways (Table S2) enriched in the gene targeted by the 24 upregulated miRNAs (Table 1) based upon DIANA-mirpath (Table S1). Interestingly, 15 out of 19 pathways in Table 3 are also included in Table S2. Thus, pathways targeted by up/downregulated miRNAs are largely overlapped. Therefore, it reveals that upregulated miRNAs, as well as downregulated miRNAs, play an important role in investigating ALS.

### 3.3. The Role of TDP-43

A previous study revealed that TDP-43 might mediate aberrant miRNA expression and ALS progression [27]. In addition, TDP-43 is largely related to enriched pathways such as AMPK pathway [25]. In addition, TDP-43 was suggested to be a part of a protein complex that processes miRNAs [28,29]. Especially, miR-663 as well as let-7b, both of which are in the identified 107 miRNAs, were up and downregulated, respectively, in the TDP-43 knock out culture cell [30]. Thus, it is not surprising that TDP-43 mediates observed aberrant expression of miRNAs.

Moreover, TDP-43 is believed to play potential roles in ubiquitin-mediated proteolysis [19]. The tight relationship between ECM and TDP-43 was also reported [31]. Enrichment analysis of crosslinking and immunoprecipitation (CLIP) identified TDP-43 target genes deregulated in ALS includes gene ontology (GO) biological process (BP) term cell adhesion [32,33]. Activation of the TGFβ/Smad signaling system is protective against the aggregate formation of cytoplasmically mislocalized TDP-43 [33].

Most interestingly, TDP-43 was reported not to cause sALS [34], which is coincident with the fact that we could not distinguish between sALS and other categories in the present study. Although Freischmidt et al. [27] once identified aberrant serum TDP-binding miRNA expression between normal control and sALS, the smallest raw (non-adjusted) *p*-value that they identified in serum miRNAs was 0.015 while they tested as many as ten miRNAs. This means, adjusted *p*-values are not regarded to be significant. Thus, their findings are not so reliable.

### 3.4. miRNAs Related to ALS

The KEGG pathways were identified using the enrichment of genes targeted by the 107 selected miRNAs. In addition to the pathway analysis, some of the selected miRNAs were previously reported to be related to ALS; miR-1290 and miR-1246 were indicated to be top down-regulated miRNAs in ALS patients [35]. The receiver operator characteristic (ROC) curve analyses revealed high diagnostic accuracy of ALS for the upregulated miR181a-5p, and this miRNA may be used as a prognostic biomarker and as an indicator of disease progression of ALS [36]. miR-4701 and miR-4485 were identified with significantly lower expression levels and significantly higher expression levels in the sALS group compared with healthy controls, respectively [37]; miR-455 and miR-26a are reduced in ALS compared to controls [38]; miR-23a was increased in skeletal muscle of ALS patients [39]; miR-146a* and miR-16-2 were dysregulated in sALS [40]; miR-22 was identified upregulation in mouse model [41]; miR-1825 was significantly down-regulated in ALS patients’ plasma [42]; miR-760, miR-744, miR-324, miR-24, miR-93, miR-17, miR-92a, miR-221, miR-103 and miR-107 were investigated in the study of identifying a complete set of miRNAs that interact with genes involved in ALS manifestation [43].

## 4. Materials and Methods

### 4.1. PCA Based Unsupervised FE

The details of the PCA based unsupervised FE method are available in the previous studies [8,9,10,11]. We simply illustrate the steps and R codes to perform this analysis.

### 4.2. Procedure 1

Step 1. Apply the R code “prcomp” on the expression profile matrix to obtain principal component (PC) loading.
Step 2. Apply the R code “lm” to calculate *p*-values for the PC loading. *p*-values are adjusted by the R code “p.adjust”. We select the PC loading with an adjusted *p*-value less than 0.05. In this case, the adjusted *p*-value of the second PC loading is less than 0.05.
Step 3. Based on the second PC score, apply the R code “pchisq” to calculate the *p*-values for miRNAs. *p*-values are adjusted by the R code “p.adjust”. In this case, 107 miRNAs with an adjusted *p*-value less than 0.01 are selected.
Step 4. Apply the R code “prcomp” on the expression profile matrix of the 107 miRNAs to obtain PC loading.
Step 5. Apply the R code “lm” to calculate *p*-values for the PC loading. *p*-values are adjusted by the R code “p.adjust”. We select the PC loading with a *p*-value less than 0.05. In this case, the *p*-values of the first, the third, the fourth and the eighth PC loadings are less than 0.05.
Step 6. Based on the four PC loadings, apply the R code “lda” (Linear Discriminate Analysis) to classify the 53 samples to four categories.

### 4.3. Linear Discriminant Analysis with PC Scores Computed Using the Selected miRNAs

**Figure 1:**
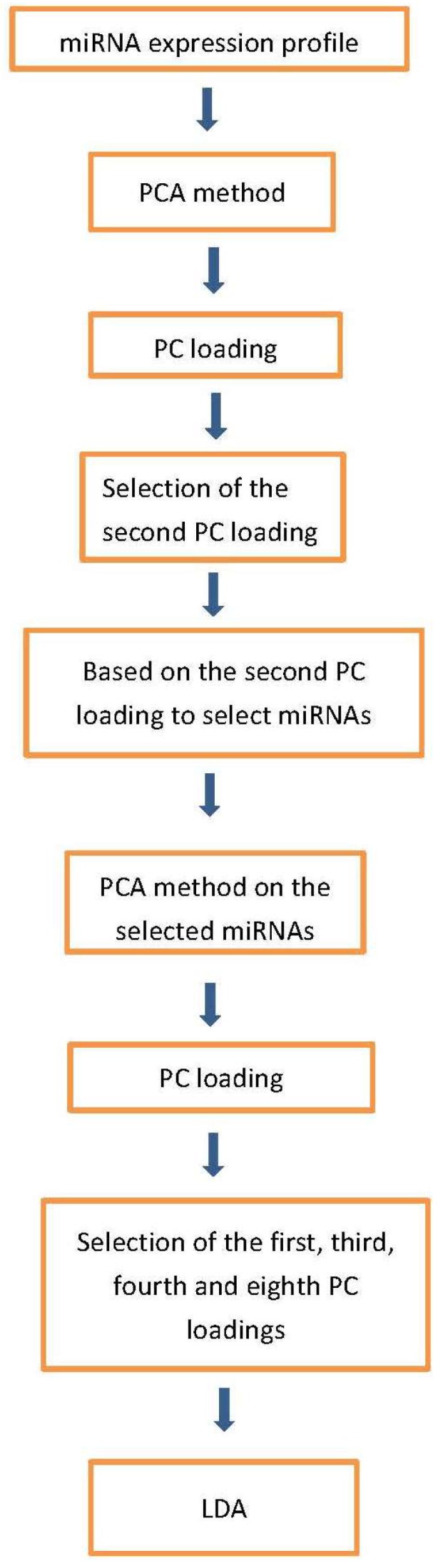
Flowchart of analyses performed in this paper.

**Figure 2:**
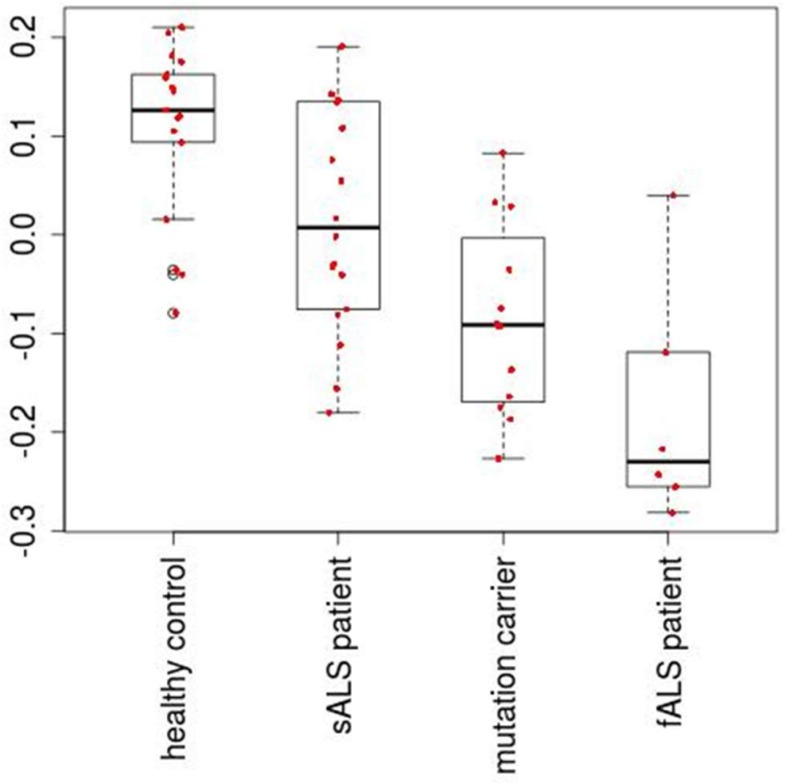
The second PC loading, which is associated with adjusted *p*-valued 2.2 × 10^−^^5^.

**Figure 3:**
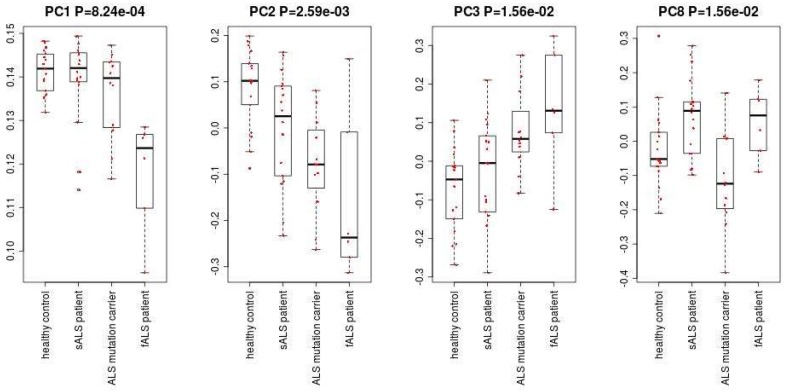
The first, the third, the fourth and the eighth PC loadings.

Figure 1 shows the flowchart of analysis performed in this paper. By Steps 1 and 2 of Procedure 1, the second PC loading is associated with significant distinction among four classes (Figure 2). The results show that healthy controls class and fALS class were most distinct. ALS mutation carrier class and sALS class were between these two where sALS class is closer to healthy control class and ALS mutation carrier class is closer to fALS. After the 107 miRNAs (Table S3) are selected by Steps 3 to 5 of Procedure 1, the first, the third, the fourth and the eighth PC loadings are associated with significant distinction among four classes (Figure 3). Then the fifty three samples are discriminated into four classes using the method of linear discriminate analysis.

**Figure 4:**
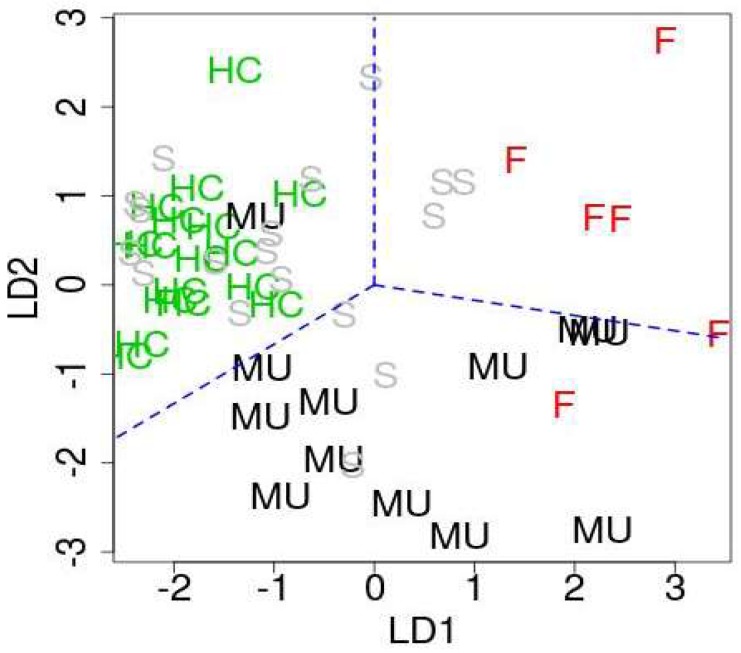
The first and second discriminate functions. HC: healthy controls, MU: ALS mutation carrier, F: fALS patients, S: sALS patients. Blue broken lines are only for a guide for eyes in order to emphasize that HC, MU, and F are well separated.

In order to identify downregulated miRNAs in ALS patients among 107 miRNAs, we compare them with the original study [12]. Then, we have found 27 significant intersections between 107 miRNAs and 33 miRNAs identified as downregulated in the original study [12] (Table 1). These 27 miRNAs are considered as downregulated miRNAs in this study. On the other hands, we have noticed that most of 27 downregulated miRNAs have positive first linear discriminant function (LD1) and second linear discriminant function (LD2) (Table S3). There are many miRNAs associated with negative LD1 and LD2 in Table S3. Thus, we consider 24 miRNAs with relatively smaller (i.e., larger absolute value) LD1 and LD2 values as upregulated miRNAs (Table 1). Figure 4 shows the scatter plot of samples with respect to LD1 and LD2. As expected, healthy controls, fALS patients, and ALS mutation carriers were well separated, while sALS patients are not.

## 5. Conclusions

In this paper, we have successfully identified miRNAs that can discriminate healthy controls from ALS patients. These miRNAs were evaluated to be related to ALS either through enrichment analysis of genes targeted by miRNAs or direct relationship between ALS and selected miRNAs. This suggested that the selected miRNAs likely regulate ALS progressions. From our results, these miRNAs can discriminate patients from healthy controls with high accuracy. Thus, they can be potential prognosis miRNA biomarkers for ALS.

## Figures and Tables

**Table 1:** List of up/downregulated miRNAs in ALS patients.

**27 downregulated miRNAs**
miR-3960; miR-3665; miR-4497; miR-4787-5p; miR-4466; miR-2861; miR-638; miR-4516; miR-4532; miR-4488; miR-4508; miR-4530; miR-3196; miR-4763-3p; miR-1469; miR-3940-5p; miR-4507; miR-4707-5p; miR-1281; miR-455-3p; miR-4270; miR-1825; miR-4745-5p; miR-4734; miR-3613-5p; miR-4741; miR-3185
**24 upregulated miRNAs**
miR-26a-5p; miR-451a; miR-181a-5p; miR-151a-5p; miR-17-5p; let-7i-5p; miR-106a-5p; miR-2278; miR-99b-3p; miR-760; miR-584-5p; let-7d-5p; miR-3175; miR-4306; miR-130b-3p; miR-324-3p; miR-221-3p; miR-744-5p; miR-25-3p; miR-4485-3p; miR-378i; miR-652-3p; miR-2392; let-7a-5p

**Table 2:** Confusion table of four ALS categories. Numbers in parentheses correspond to the performances when sALS patients are excluded and discrimination was performed using three categories.

	True	Healthy Control	sALS Patient	ALS Mutation Carrier	fALS Patient
Prediction					
**healthy Control**		14 (16)	6 (-)	0 (1)	0 (0)
**sALS Patient**		3 (-)	8 (-)	2 (-)	0 (-)
**ALS Mutation Carrier**		0 (1)	2 (-)	8 (7)	1 (2)
**fALS Patient**		0 (0)	2 (-)	2 (4)	5 (4)

**Table 3:** KEGG pathways enriched in targeted genes by the 27 downregulated miRNAs in Table 1.

KEGG Pathway	*p-*Value	#Genes	#miRNAs
ECM-receptor interaction	1.45 × 10^−10^	10	3
Adherens junction *	1.21 × 10^−9^	16	6
Transcriptional misregulation in cancer *	4.39 × 10^−6^	23	6
Cell cycle *	4.39 × 10^−6^	25	8
Hippo signaling pathway *	6.77 × 10^−6^	24	9
Oocyte meiosis *	3.39 × 10^−5^	18	5
TGF-beta signaling pathway *	0.000314	13	2
Protein processing in endoplasmic reticulum *	0.000971	25	8
Pantothenate and CoA biosynthesis	0.001459	3	5
RNA transport *	0.002423	23	7
Focal adhesion *	0.005153	29	8
Ubiquitin mediated proteolysis *	0.008498	21	8
Colorectal cancer *	0.010697	10	7
2-Oxocarboxylic acid metabolism	0.017091	3	3
mRNA surveillance pathway *	0.023398	15	4
AMPK signaling pathway *	0.023398	19	6
Proteoglycans in cancer *	0.023929	23	6
Regulation of actin cytoskeleton *	0.03352	23	6
Spliceosome	0.03352	18	7

* denotes these pathways are targeted by both upregulated and downregulated miRNAs.

## Supplementary Materials

Supplementary materials can be found at http://www.mdpi.com/1422-0067/19/5/1318/s1.
