# Supplementary material for: Exploring microRNA Biomarker for Amyotrophic Lateral Sclerosis
Source: Int J Mol Sci. 2018 Apr 28;19(5):1318. doi: 10.3390/ijms19051318 (PMC5983737; doi:10.3390/ijms19051318)
Supplement: Supplementary file 1 [file ijms-19-01318-s001.zip › ijms-293932-s/Table S2.pdf]

Table S1. KEGG pathways enriched in targeted genes by the 24 upregulated miRNAs in **Error! Reference source not found.**. * denotes these pathways are targeted by both upregulated and downregulated miRNAs.

KEGG pathway	p-value	#genes	#miRNAs
Proteoglycans in cancer	1.67E-19	141	22
Renal cell carcinoma	1.26E-09	56	22
Protein processing in endoplasmic reticulum*	1.89E-09	118	22
Viral carcinogenesis	3.38E-09	135	23
Adherens junction*	3.60E-09	59	21
TGF-beta signaling pathway*	5.27E-09	59	23
Hippo signaling pathway*	2.34E-08	97	23
Cell cycle*	5.67E-08	90	22
Bacterial invasion of epithelial cells	1.30E-07	57	20
Fatty acid metabolism	6.05E-07	27	19
Lysine degradation	6.09E-07	33	19
Colorectal cancer*	6.29E-07	48	21
p53 signaling pathway	9.69E-07	53	20
N-Glycan biosynthesis	1.51E-06	35	18
Biosynthesis of unsaturated fatty acids	1.77E-06	16	16
Thyroid hormone signaling pathway	3.27E-06	81	22
Prostate cancer	3.34E-06	65	22
Shigellosis	4.34E-06	48	22
FoxO signaling pathway	4.34E-06	92	22
Ubiquitin mediated proteolysis*	4.50E-06	97	22
Endometrial cancer	5.19E-06	41	20
Glycosaminoglycan biosynthesis - keratan sulfate	6.01E-06	11	12
Estrogen signaling pathway	6.61E-06	66	22
Pathways in cancer	6.61E-06	236	23
Glioma	9.71E-06	45	20
Prion diseases	1.10E-05	18	18
Pancreatic cancer	1.21E-05	49	21
Hepatitis B	1.81E-05	92	23
Transcriptional misregulation in cancer*	1.83E-05	108	23
Oocyte meiosis*	2.20E-05	74	21
Endocytosis	3.54E-05	137	21
Regulation of actin cytoskeleton*	3.76E-05	129	22
Signaling pathways regulating pluripotency of stem cells	5.23E-05	88	21
Focal adhesion*	5.73E-05	130	22

Chronic myeloid leukemia	6.68E-05	52	21
Progesterone-mediated oocyte maturation	9.03E-05	62	21
Acute myeloid leukemia	0.000236	41	21
Neurotrophin signaling pathway	0.000392	79	22
Axon guidance	0.000564	73	21
Bladder cancer	0.000649	30	18
mRNA surveillance pathway*	0.000649	62	21
mTOR signaling pathway	0.000935	43	21
Thyroid cancer	0.001001	21	19
Sphingolipid signaling pathway	0.001001	73	21
MAPK signaling pathway	0.001631	144	22
Non-small cell lung cancer	0.001647	37	20
Insulin signaling pathway	0.002729	87	22
Small cell lung cancer	0.002857	56	23
Fatty acid biosynthesis	0.00446	6	13
Huntington's disease	0.004742	107	22
Ribosome	0.005396	83	20
RNA transport*	0.007227	99	22
Epstein-Barr virus infection	0.007274	122	23
Melanoma	0.007358	45	20
Arrhythmogenic right ventricular cardiomyopathy (ARVC)	0.007778	36	20
Salmonella infection	0.007778	54	22
Circadian rhythm	0.00844	23	19
HIF-1 signaling pathway	0.011101	66	22
Steroid biosynthesis	0.012579	13	14
Central carbon metabolism in cancer	0.012752	42	20
Rap1 signaling pathway	0.013498	117	22
ErbB signaling pathway	0.014677	55	22
TNF signaling pathway	0.015772	70	21
PI3K-Akt signaling pathway	0.017214	187	23
Other types of O-glycan biosynthesis	0.022656	16	15
Platelet activation	0.023844	74	22

Non-alcoholic fatty liver disease (NAFLD)	0.024311	89	22
Fc gamma R-mediated phagocytosis	0.026365	55	21
VEGF signaling pathway	0.026932	39	20
AMPK signaling pathway*	0.026932	77	22
RNA degradation	0.02877	50	22
Fatty acid elongation	0.030304	15	15
Gap junction	0.030378	51	20
Epithelial cell signaling in Helicobacter pylori infection	0.033539	42	16
Prolactin signaling pathway	0.038056	45	20
Apoptosis	0.041464	51	20
Choline metabolism in cancer	0.042193	61	21
Dorso-ventral axis formation	0.047968	19	19
